# Ertugliflozin improves animal behaviours associated with oxidative stress and inflammation in a BTBR *T* + *Itpr3tf/J* mouse model of autism

**DOI:** 10.1093/braincomms/fcag083

**Published:** 2026-03-11

**Authors:** Xiaona Wang, Zhengqin Zhao, Limin Sun, Chao Gao, Li Wang, Daoqi Mei, Chanjuan Hao, Shuai Zhao, Xingxue Yan, Jing Liu, Lei Liu, Bin Guo, Yaodong Zhang

**Affiliations:** Henan Key Laboratory of Genetic and Developmental Disorders, Henan Children's Neurodevelopment Engineering Research Center, Children's Hospital Affiliated to Zhengzhou University, Henan Children's Hospital, Institute of Children's Health, Henan Academy of Medical Sciences, Zhengzhou, 450018, China; Department of Nuclear Medicine, Affiliated Hospital of Guangdong Medical University, Zhanjiang, Guangdong, China; Shanghai Institute of Microsystem and Information Technology, Shanghai, China; Department of Rehabilitation, Children's Hospital Affiliated to Zhengzhou University, Zhengzhou, China; Department of Neurology, Children's Hospital Affiliated to Zhengzhou University, Zhengzhou, China; Department of Neurology, Children’s Hospital Affiliated to Soochow University, Suzhou, China; Beijing Key Laboratory for Genetics of Birth Defects, Beijing Pediatric Research Institute, MOE Key Laboratory of Major Diseases in Children, Genetics and Birth Defects Control Center, Beijing Children’s Hospital, Capital Medical University, National Center for Children’s Health, Beijing, China; Henan Key Laboratory of Genetic and Developmental Disorders, Henan Children's Neurodevelopment Engineering Research Center, Children's Hospital Affiliated to Zhengzhou University, Henan Children's Hospital, Institute of Children's Health, Henan Academy of Medical Sciences, Zhengzhou, 450018, China; Henan Key Laboratory of Genetic and Developmental Disorders, Henan Children's Neurodevelopment Engineering Research Center, Children's Hospital Affiliated to Zhengzhou University, Henan Children's Hospital, Institute of Children's Health, Henan Academy of Medical Sciences, Zhengzhou, 450018, China; Henan Key Laboratory of Genetic and Developmental Disorders, Henan Children's Neurodevelopment Engineering Research Center, Children's Hospital Affiliated to Zhengzhou University, Henan Children's Hospital, Institute of Children's Health, Henan Academy of Medical Sciences, Zhengzhou, 450018, China; Henan Key Laboratory of Genetic and Developmental Disorders, Henan Children's Neurodevelopment Engineering Research Center, Children's Hospital Affiliated to Zhengzhou University, Henan Children's Hospital, Institute of Children's Health, Henan Academy of Medical Sciences, Zhengzhou, 450018, China; Physiology and Psychology Section, Ningxia Regional Key Laboratory of Integrated Traditional Chinese and Western Medicine for Prevention and Treatment of Regional High Incidence Disease, Ningxia Medical University, Yinchuan City 750004, China; Henan Key Laboratory of Genetic and Developmental Disorders, Henan Children's Neurodevelopment Engineering Research Center, Children's Hospital Affiliated to Zhengzhou University, Henan Children's Hospital, Institute of Children's Health, Henan Academy of Medical Sciences, Zhengzhou, 450018, China

**Keywords:** autism spectrum disorder, ertugliflozin, oxidative stress, inflammation, microglia

## Abstract

Autism spectrum disorder is a neurodevelopmental condition typified by difficulties in social interactions, repetitive and restricted behaviour and heightened anxiety. Increasing evidence suggests that oxidative stress and neuroinflammatory processes are crucial in the development of these behavioural abnormalities. Ertugliflozin, a sodium-glucose cotransporter-2 inhibitor approved by the FDA for treating type 2 diabetes mellitus, has also been reported to exert antioxidant and anti-inflammatory effects. BTBR *T* + *Itpr3tf/J* (BTBR) mice are widely used as a preclinical model of autism spectrum disorder, as they show core autism-like behavioural features. The present study investigated whether ertugliflozin could ameliorate autism spectrum disorder-like behaviour in BTBR mice and explored the associated mechanisms. It was found that ertugliflozin treatment significantly improved social interaction while reducing repetitive behaviours and anxiety-like responses compared with untreated BTBR mice. Ertugliflozin (20 mg/kg/day), administered orally, reduced neuronal loss in the CA1 region of the hippocampus and the prefrontal cortex. In addition, ertugliflozin reduced oxidative stress, as demonstrated by decreased malondialdehyde levels, restoration of glutathione content and improved activities of superoxide dismutase and catalase. A significant suppression of inflammatory cytokines accompanied these biochemical improvements. Furthermore, ertugliflozin significantly inhibited microglial activation in BTBR mice. Collectively, the findings indicate that ertugliflozin alleviates autism spectrum disorder-like behavioural deficits in BTBR mouse models, at least in part, by reducing oxidative stress and neuroinflammation. This study highlights ertugliflozin as a potential therapeutic candidate for the management of autism spectrum disorder.

## Teaser

Wang *et al*. demonstrate that ertugliflozin ameliorates core autism-like behaviour in BTBR T + Itpr3tf/J mice, improving social interaction while reducing repetitive behaviours and anxiety. These improvements correlate with attenuated neuronal loss, suppressed oxidative stress and reduced neuroinflammation. Our findings support ertugliflozin’s potential as a candidate for treating autism spectrum disorder.

## Graphical Abstract

**Graphical Abstract:**
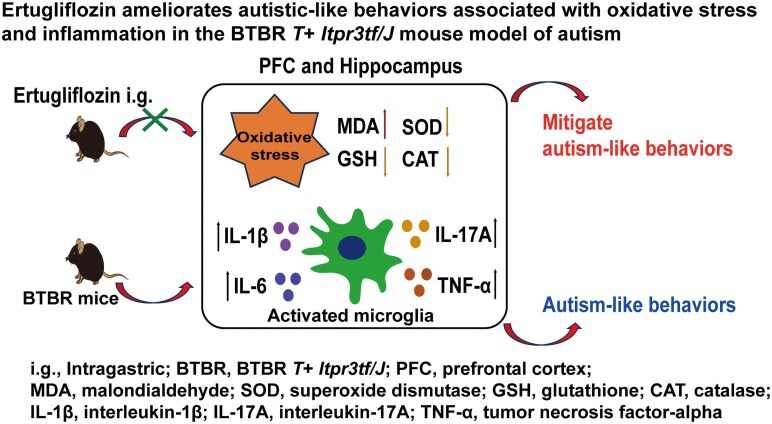

## Introduction

Autism spectrum disorder is a neurodevelopmental condition associated with decreased social interaction, repetitive conduct and anxiety-like symptoms.^1,2^ Recent reports of a much greater prevalence rate for autism spectrum disorder have made it a global health concern.^3^ Autism spectrum disorder is a complex condition caused by both hereditary and environmental factors.^4^ However, there are few successful autism spectrum disorder treatments and intervention techniques, and its precise pathophysiology is still unknown.

A body of data suggests that oxidative inflammation and immunological activation in the prefrontal cortex (PFC) and hippocampus are implicated in abnormal neurodevelopment, which underlies the pathophysiology of autism spectrum disorder disorder.^5-8^ Disruption of the balance between ROS generation and the antioxidant system leads to oxidative stress, which can cause cytotoxicity by damaging proteins and nucleic acids.^3^ Oxidative stress can damage proteins and nucleic acids, resulting in cytotoxicity.^3,9^ Meanwhile, neuroinflammation, particularly microglial activation, triggers a cascade of inflammatory responses that exacerbate neuronal dysfunction.^9^These processes disrupt normal brain development, contributing to behavioural disturbances observed in autism spectrum disorder.^10^ In this concept, researchers affirmed that some agents that have beneficial influences and ameliorate autism spectrum disorder-like symptoms in animal models through attenuation of oxidative stress and neuroinflammation.^11-14^

Diabetes mellitus, ischaemic stroke, Alzheimer’s disease and epilepsy have all been shown to benefit from sodium-glucose cotransporter 2 (SGLT2) inhibitors.^15^ Ample evidence indicates that SGLT2 inhibitors possess neuroprotective properties, attributable to their antioxidant and anti-inflammatory activities.^16-18^ SGLT2 inhibitors encompass canagliflozin and ertugliflozin. Specifically, canagliflozin mitigated oxidative stress status and autism spectrum disorder-like features in valproic acid-treated rats.^19^ Ertugliflozin is a drug used to treat type 2 diabetes, particularly in those with heart failure.^20,21^ Ertugliflozin was shown to reduce pro-inflammatory cytokine levels.^17^ Despite this strong evidence, it is still unclear if ertugliflozin modulates oxidative stress and neuroinflammation to improve behavioural traits linked with autism spectrum disorder.

This BTBR *T* + *Itpr3tf/J* (BTBR) animal model is considered a translational tool to assess potential therapies for autism spectrum disorder.^9^ BTBR mice display behavioural patterns thought to be associated with core domains of autism spectrum disorder.^22^ The BTBR mouse shows increased oxidative stress and aberrant immune responses that may contribute to its characteristic phenotypes in autism spectrum disorder.^12,23-26^ This study explored the potential impact of ertugliflozin on autism spectrum disorder-like behavioural disturbances in BTBR mice. Moreover, we investigated its underlying mechanisms, particularly its effects on oxidative stress, inflammatory mediators and microglial activation in the hippocampus and PFC. The results of this research could unveil novel perspectives on the therapeutic possibilities of ertugliflozin in mitigating the autism spectrum disorder-associated symptoms and provide valuable insights into the future understanding in the fields ertugliflozin of pharmacology.

## Materials and methods

### Animals

All animal experimental procedures were performed in accordance with the National Institutes of Health Guide for the Care and Use of Laboratory Animals and were approved by the Animal Ethics Committee of Zhengzhou University. Male BTBR *T* + *Itpr3tf/J* (BTBR; JAX #002282) mice were received from the Jackson Laboratory (USA), whereas C57BL/6J mice were procured from Zhengzhou University. Experiments were carried out using 8-week-old male mice weighing 22–24 g. The animals were kept in groups of three to four per cage with a conventional 12-h light/dark cycle and constant humidity and temperature (22 ± 2°C, 55 ± 2%). Throughout the trial, food and water were freely supplied. Special care was taken to minimize animal pain and discomfort throughout all experimental procedures.

### Animal grouping and drug administration

Based on previous reports,^5,27^ BTBR mice were allocated to four groups using computer-generated random numbers: the control group (C57), the ertugliflozin group (C57 + Ert), the model group (BTBR) and the ertugliflozin treatment group (BTBR + Ert). The two ertugliflozin treatment groups were administered an ertugliflozin solution once a day by gavage beginning on Postnatal Day 60. The dosing regimen for ertugliflozin (20 mg/kg/day) was established based on previously published studies.^19^ The C57 and BTBR groups were administered the same volume of saline by gavage once daily. For 8 weeks in a row, the treatments were given every day. A 0.2 mg/ml solution of ertugliflozin (MedChemExpress, Shanghai, China; Cat#HY-15461) was made by diluting it in 5% DMSO with 0.9% saline.

Thirty minutes after the last drug delivery, mice were put through a series of behavioural assessments. Following the behavioural tests, the animals were euthanized (*n* = 10 per group). The brains were harvested, followed by dissection of the PFC and hippocampus according to the mouse atlas, for the following tests.

### Sociability and social novelty testing

Social behaviour in mice was evaluated through the three-chamber social interaction test, as described previously.^28^ The testing apparatus comprised a rectangular Plexiglas box (60 × 40 × 22 cm) consisting of three compartments (each measuring 40 × 20 × 22 cm) by removable partitions, with grey walls throughout. Illumination across the entire apparatus was maintained at 5 lx. During the habituation phase, mice were introduced into the central compartment and permitted to acclimate for 10 min. Test mice were matched by strain and sex with unfamiliar stimulus mice, with no prior contact between them. For the sociability session, an unfamiliar male mouse (Stranger 1) was positioned inside a wire cage (diameter, 8 cm) in one side chamber.

In comparison, an identical empty cage was installed in the opposing chamber. The animal explored the three chambers for 10 min. In the ensuing social preference phase, a further unknown mouse (Stranger 2) was introduced into the empty cage, and the subject mouse was granted an extra 10 min of free exploration. Using the camera-assisted VisuTrack software (Xinruan Information Technology Co., Ltd., Shanghai, China), the time within each chamber, smelling and direct engagement with unfamiliar mice were automatically recorded.

Sociability was assessed by determining a discriminating index for each animal as (time spent with unfamiliar Mouse 1—time spent with the stranger object) divided by (total time spent with unfamiliar Mouse 1 and the novel object). As previously indicated, the discriminating index (time spent with novel Mouse 2—time spent with stranger mouse 1)/(total time spent with stranger Mouse 1 and novel Mouse 2) was calculated to determine preference for social novelty.^29,30^ An investigator who was blind to the mice treatment calculated all of the scores.

### Self-grooming

Self-grooming tests were used to check for compulsive and repetitive behaviour, which is another common autism spectrum disorder characteristic.^31,32^ The conditioning chamber was rectangular (40 × 20 × 10 cm) and included bedding (2 cm deep) and a light of ∼30 lx. The duration and number of times spent grooming were recorded for 20 min. Grooming behaviours included face cleaning, head and ear scratching and rubbing and grooming of the overall body.

### Marble-burying

The marble-burying was assessed according to published protocols.^1^ For 10 min, the animals were placed in a clean cage (30 × 20 × 15 cm) loaded with about 6 cm of wood chips. After that, a 3 × 4 array of 16 clean glass marbles was uniformly distributed. The marbles were given to the mice for 30 min. Marbles were considered buried when 67% or more of their size was covered with bedding. Testing was carried out under dim light circumstances (∼30 lx).

### Open-field test

As in our previous publications, open-field tests were conducted.^33^ The animals were put in the centre of an open-field box (50 × 50 × 45 cm). The amount of time in the central region (25 × 25 cm) and the distance moved were recorded for 30 min. The testing room had roughly 30 lx of lighting.

### Elevated plus maze

The EPM device contained two open arms (75 × 58 × 6 cm) on opposite sides and two opposing closed arms (75 × 58 × 6 cm). The central part, a square measuring 6 × 6 cm, linked the arms. The device was set at a height of 60 cm from the ground. Animals were positioned in the centre, faced towards the open arms, and could investigate for 10 min. The overall distance covered and time within the open arms were among the parameters assessed. The illumination of the room was ∼30 lx.

### Nissl staining

Frozen tissue sections (30 μm; *n* = 10 per group) were air-dried at room temperature. The slides were sequentially dehydrated in 100% ethanol, 95% ethanol and distilled water and subsequently stained with 0.1% cresyl violet at 56°C for 10 min, followed by differentiation in an HCl/95% ethanol solution (1:50) for 2 min. Subsequently, sections were dehydrated in a gradient of ethanol (70, 90, 100%), incubated in xylene for 2 min and mounted on coverslips. Images of the PFC and CA1 regions of the hippocampus were captured using light microscopy (Nikon Instruments, Osaka, Japan).

### ELISA

BCA assays (Solarbio, Beijing, China) were initially used to assess the protein levels in mouse brain tissue supernatants (*n* = 8 per group for oxidative stress indices; *n* = 10 per group for cytokine measures). Oxidative stress markers were then evaluated using commercially available kits. Catalase (CAT) activity was assessed using a kit (Cat#A007–1-1; Nanjing Jiancheng Bioengineering Institute), as was superoxide dismutase (SOD) (Cat#A001-1-2; Nanjing Jiancheng Bioengineering Institute). Moreover, glutathione (GSH) levels were quantified using a kit (Cat#A006-1-1; Nanjing Jiancheng Bioengineering Institute), while malondialdehyde (MDA) content was also measured with a kit (Cat#A003-1-2; Nanjing Jiancheng Bioengineering Institute).

Levels of inflammatory cytokines were measured as directed. ELISA kits from Multi Sciences (Hangzhou, China; Cat#EK282 and Cat#EK206, respectively) were used to measure IL-6 and TNF-α. Interleukin-1β (IL-1β) concentrations were measured with a corresponding ELISA kit (Cat#EK201B; Multi Sciences), whereas IL-17A levels were assessed employing an ELISA kit obtained from Cusabio Biotech Co., Ltd. (Cat#CSB-E04608 m).

### Immunohistochemical (IHC) assay

IHC methods were used in this study, as we previously detailed.^33^ Animals (n = 10) were deeply anaesthetized with 1% pentobarbital sodium before transcardial perfusion with 4% paraformaldehyde for fixation. The brains were immediately harvested, post-fixed overnight at 4°C and kept in 30% sucrose. Coronal brain slices (40 μm thickness) were produced using a vibratome (Leica, Germany) in 0.1 M PBS. Free-floating sections were first treated with a blocking buffer consisting of 5% donkey serum, 2.5% BSA and 0.5% Triton X-100 in PBS. The tissues were then incubated overnight at 4°C with an anti-ionized calcium-binding adaptor molecule 1 (Iba1) primary antibody (1:500; Wako, Tokyo, Japan; Cat#019-19741). Sections were cleaned and then exposed to donkey anti-rabbit IgG (1:600; Invitrogen, USA; Cat#A-10042) conjugated with Alexa Fluor 568 for 2 h at room temperature. Nuclear counterstaining was done for 30 min with DAPI (1:1000; Merck, USA; Cat#MBD0015). Sections were washed three times in PBS (10 min each), mounted in a glycerol:PBS mixture (7:3) and examined with an LSM980 confocal microscope (Carl Zeiss). Quantification of activated microglia in the PFC and hippocampus was conducted with the ImageJ platform.

### Statistical analysis

Data were analysed using SPSS 26.0 and are shown as mean ± standard error of the mean (SEM). T-tests or two-way analysis of variance (ANOVA) were used for group comparisons, and Tukey’s post hoc multiple-comparison test was used when necessary. Each value represents an independent experimental observation. Statistical significance was defined as a *P* < 0.05.

## Results

### Ertugliflozin treatment enhanced social interaction and novelty responses in BTBR mice

**Figure 1:**
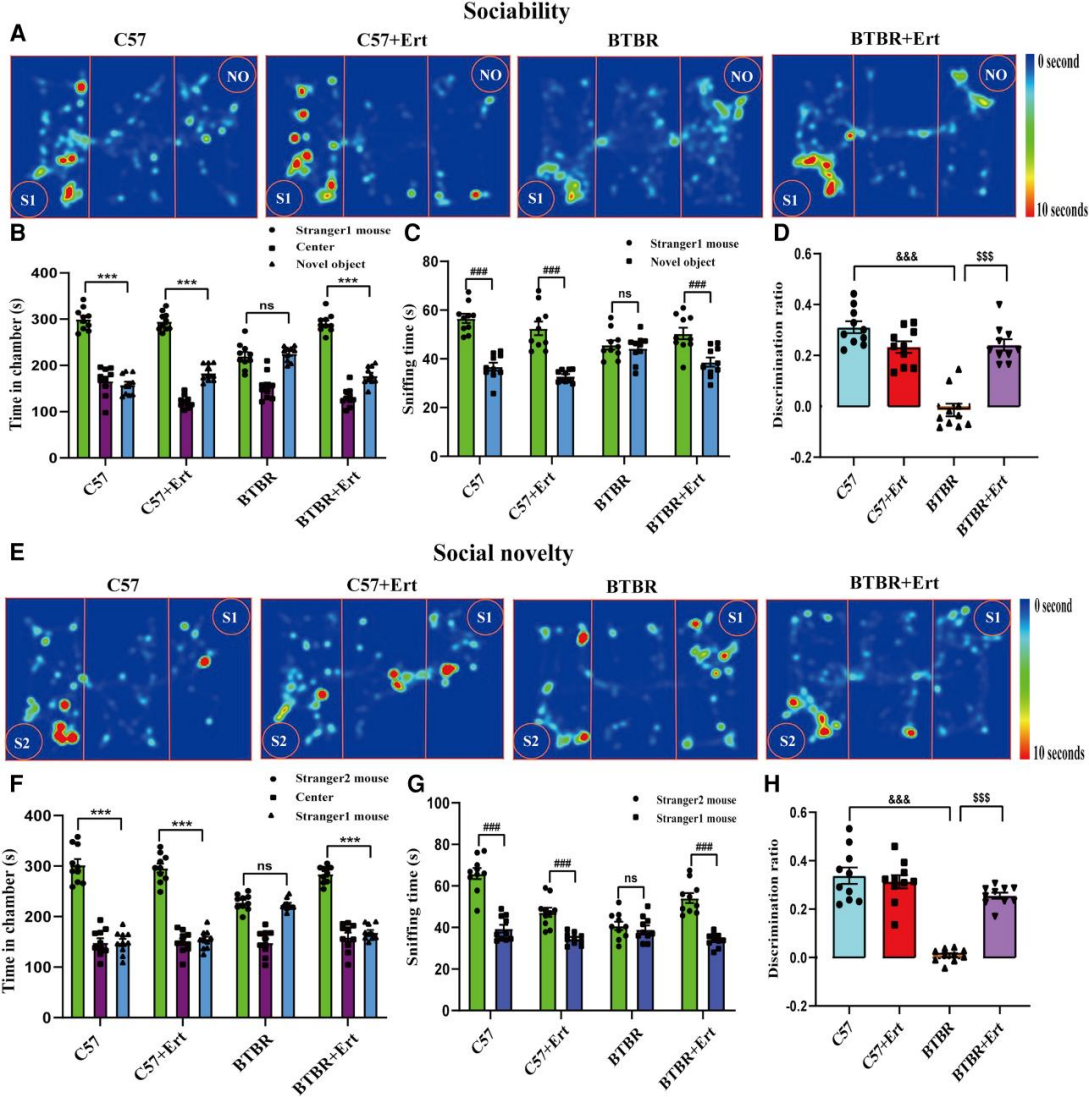
**Administration of ertugliflozin alleviates social impairments in BTBR mice**. (**A**) Heatmaps obtained from the three-chamber sociability test, with warmer colours (red) indicating longer exploration time. ‘NO’ denotes the novel object, and ‘S1’ indicates the unfamiliar mouse. (**B–D**) Quantification of time spent in each chamber (**B**), duration of sniffing behaviour (**C**) and discrimination index (**D**) during the social interaction phase. (**E**) Representative heat maps from the social novelty preference test, where warmer colours reflect increased exploration. ‘S1’ represents the familiar mouse, and ‘S2’ indicates the newly introduced stranger mouse. (**F–H**) Time spent in each compartment (**F**), total interaction duration (**G**) and social preference index (**H**) during the social novelty session. Data are expressed as mean ± SEM, with each symbol indicating an individual mouse (*n* = 10 per group). Statistical analyses for panels **B**, **C**, **E** and **F** were performed using t-tests, while panel **H** was analysed by two-way ANOVA followed by Tukey’s post hoc test. ***P < 0.001 for novel mouse versus novel object; ^###^*P* < 0.001 for novel mouse versus familiar mouse; ^&&&^*P* < 0.001 for BTBR versus C57 groups; ^$$$^*P* < 0.001 for BTBR + Ert versus BTBR groups; ns indicates no significant difference.

Both the C57 and C57 + Ert groups spent considerably longer in the area with the unfamiliar mouse (S1) than in the empty cage (Novel object, NO) during the social interaction test, as seen in Fig. 1A-D (both *P* < 0.001). In comparison, BTBR mice tended to stay in the empty chamber rather than interacting with the stranger mouse (*P* < 0.001). Similarly, BTBR mice showed no social preference, with no discernible difference in exploration between the novel mouse and the empty compartment. In comparison, C57 and C57 + Ert mice (both *P* < 0.001) spent markedly longer sniffing the strange mouse relative to the empty cage (*P* > 0.05). Furthermore, compared with C57 controls, BTBR mice showed a considerably reduced discrimination index (*P* < 0.001). Importantly, ertugliflozin treatment significantly reversed these issues in BTBR mice, as evidenced by increased time spent in the stranger mouse chamber (*P* < 0.001), enhanced sniffing of the novel animal (*P* < 0.001) and improved discrimination index (*P* < 0.001). These findings collectively indicate that ertugliflozin significantly enhances social interaction behaviour.

Compared to the familiar S1 animal, C57 and C57 + Ert mice stayed longer in the chamber with a stranger mouse (S2) during the social novelty preference session (Fig. 1E-H) (both *P* < 0.001). Relative to the familiar mouse, C57 and C57 + Ert mice sniffed the new animal for longer (both *P* < 0.001). Relative to the new mouse, BTBR mice showed no preference for investigating the area with the familiar mouse (*P* > 0.05). In comparison with a stranger mouse, BTBR mice spent the same length of time smelling and interacting with a familiar animal (*P* > 0.05). Furthermore, we observed that BTBR mice showed a substantially lower preference index than C57 mice (*P* < 0.001). Reductions in time in the novel compartment (*P* < 0.001), sniffing time (*P* < 0.001) and discrimination time (*P* < 0.001) were noted following ertugliflozin administration. In BTBR mice, these effects were significantly improved, suggesting that ertugliflozin restores changed preferences for social novelty.

### Ertugliflozin diminishes stereotyped/repetitive and anxiety-like behaviours

**Figure 2:**
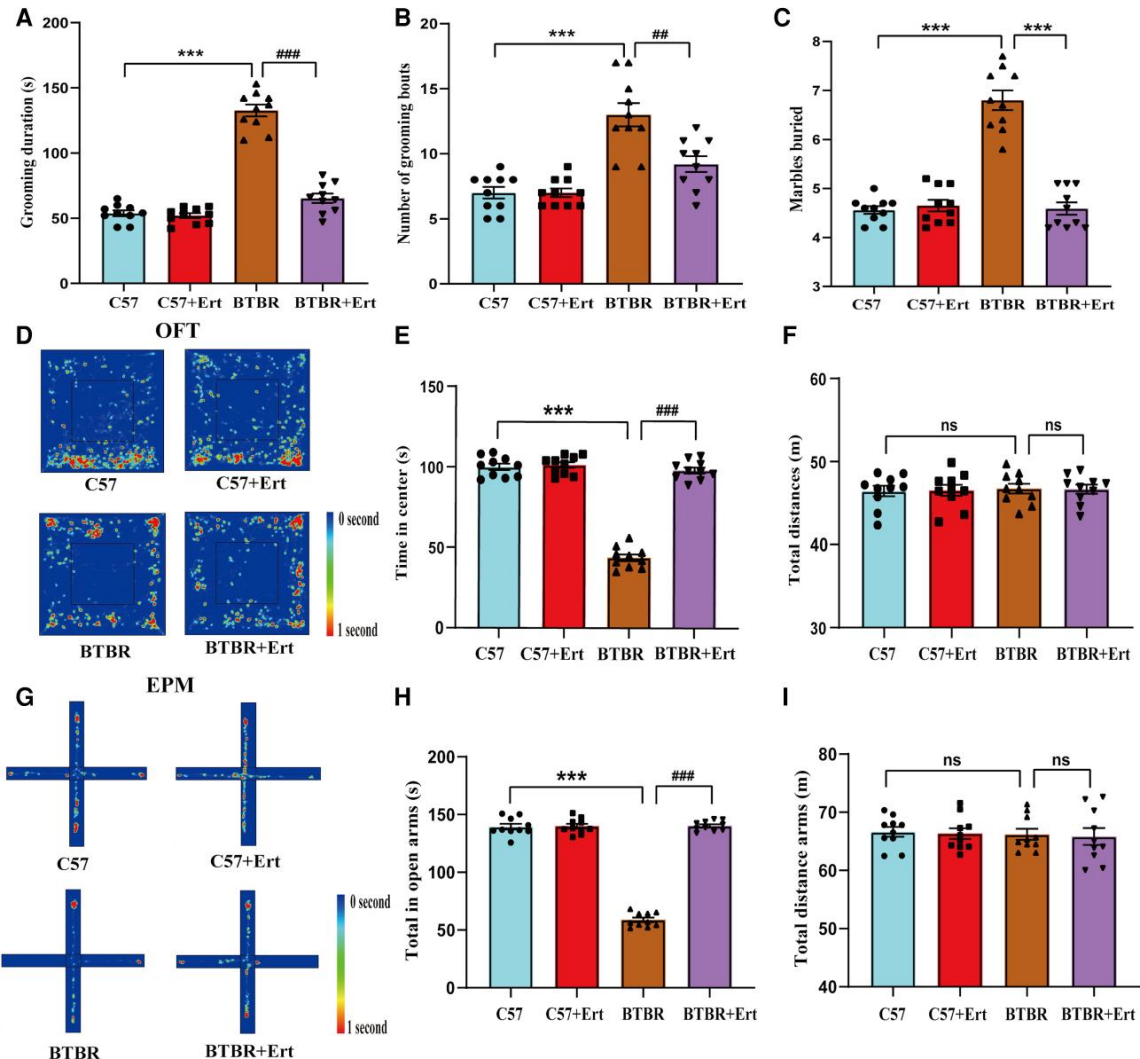
**Ertugliflozin treatment relieved high levels of repetitive behaviours and anxiety in BTBR mice.** (**A-B**) Self-grooming behaviour of experimental mice, including total grooming duration (**A**) and frequency of grooming bouts (**B**). (**C**) Quantification of marbles buried in the marble-burying test. (**D-F**) Behavioural outcomes were assessed using the open-field test. (**D**) Trajectory diagram during the entire 30 min. Warmer colours (red) correspond to areas where the animal spent more time exploring. (**E**) Distance in the centre region. (**F**) Quantification of the total distance. (**G-I**) Elevated plus maze test. (**G**) Trajectory diagram during a 10-min test session. Warmer colours (red) indicate areas of more exploration. (**H**) Time spent exploring the open arms. (**I**) Total distance moved. Results are expressed as the mean ± SEM. Data points represent individual mice (*n* = 10 per group). Results are statistically compared by two-way ANOVA with Tukey’s *post hoc* test. **P < 0.01, ***P < 0.001 for BTBR group versus C57 group; ^##^*P* < 0.01, ^###^*P* < 0.001 for BTBR + Ert group versus BTBR group; ‘ns’ indicates not significantly different.

It is well known that stereotyped, repetitive patterns of behaviour have been studied using the self-grooming and marble-burying tests.^24,29^ In the self-grooming assay, BTBR mice demonstrated significantly prolonged grooming duration and a higher grooming episode frequency relative to controls (both *P* < 0.001; Fig. 2A-B). In comparison, administration of ertugliflozin significantly reduced these elevated self-grooming behaviours, leading to decreases in grooming time and bout frequency (both *P* < 0.01; Fig. 2A-B). The marble-burying test consistently showed that BTBR mice buried a larger number of marbles than C57 mice (*P* < 0.01; Fig. 2C). Relative to untreated BTBR mice, ertugliflozin therapy markedly reduced the quantity of marbles buried (*P* < 0.01; Fig. 2C).

To further evaluate the ability of ertugliflozin to mitigate anxiety-associated behaviour in BTBR mice, maze tests (open-field and elevated) were conducted (Fig. 2D-I). While the overall distance walked throughout the 30-min session did not differ between groups (*P* > 0.05; Fig. 2F), BTBR mice in the open-field test spent considerably less time in the central area than control animals (*P* < 0.001; Fig. 2D-E). Treatment with ertugliflozin significantly improved the time BTBR mice spent in the centre zone relative to untreated BTBR mice (*P* < 0.001; Fig. 2D-E), without affecting overall locomotor activity (*P* > 0.05; Fig. 2F).

Consistent results were obtained in the elevated plus maze test, where BTBR mice demonstrated a pronounced reduction in both the time spent and number of entries into the open arms compared with C57 mice (*P* < 0.001; Fig. 2G-H). Importantly, ertugliflozin administration significantly restored exploratory behaviour in the open arms during the 10-min testing period relative to untreated BTBR mice (*P* < 0.001; Fig. 2G-H). There were no discernible differences in the groups’ overall travel distances (*P* > 0.05; Fig. 2I). These results show that ertugliflozin reduces key behavioural abnormalities associated with autism spectrum disorder, such as repetitive behaviours and anxiety-like responses.

### Ertugliflozin improves neuronal morphology in BTBR mouse brain

**Figure 3:**
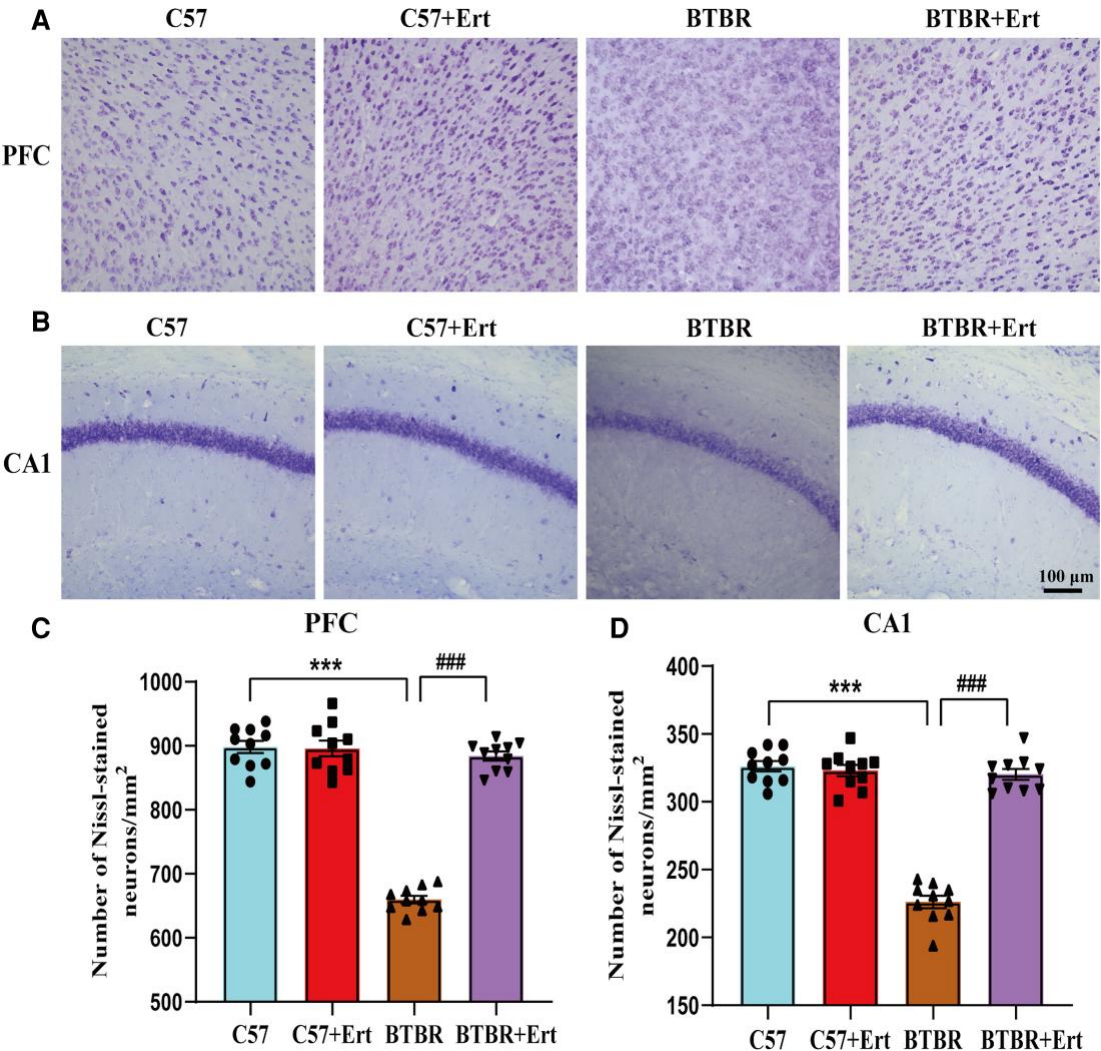
**Effects of ertugliflozin treatment on the microstructure of the PFC and CA1 area of the hippocampus**. (**A**, **C**) Images of Nissl staining (**A**) and quantification (**C**) of the number of viable neurons in the PFC. (**B**, **D**) Images of Nissl staining (**B**) and analyses of the cell number (**D**) in the CA1 area. Data are presented as mean ± SEM. Data points denote individual mice (*n* = 10 for each group). Data are statistically compared by two-way ANOVA with Tukey’s *post hoc* test. ***P < 0.001 compared to C57 mice; ^###^*P* < 0.01 compared to BTBR mice.

To evaluate pathological changes in neuronal integrity, Nissl staining was performed to assess neuronal viability. Neurons in the PFC and CA1 region of the hippocampus of C57 mice were densely arranged and showed abundant Nissl bodies with well-defined morphology (Fig. 3A-B). In comparison, BTBR mice displayed sparse neuronal distribution and irregularly shaped cell bodies in both regions. Quantitative analysis revealed marked reductions in the number of Nissl-positive neurons in BTBR brain sections relative to controls (both *P* < 0.001; Fig. 3C). Importantly, brain slices from ertugliflozin-treated BTBR mice showed prominent blue granular Nissl bodies and improved cellular organization, accompanied by a significant increase in Nissl-stained neurons relative to untreated BTBR mice (*P* < 0.001; Fig. 3D). Collectively, these findings demonstrate that ertugliflozin successfully mitigates neuronal injury in the hippocampus and PFC of BTBR mice, as represented by restored neuronal morphology and increased neuronal survival.

### Mitigating impacts of ertugliflozin on oxidative stress in the PFC and hippocampus of BTBR mice

**Figure 4:**
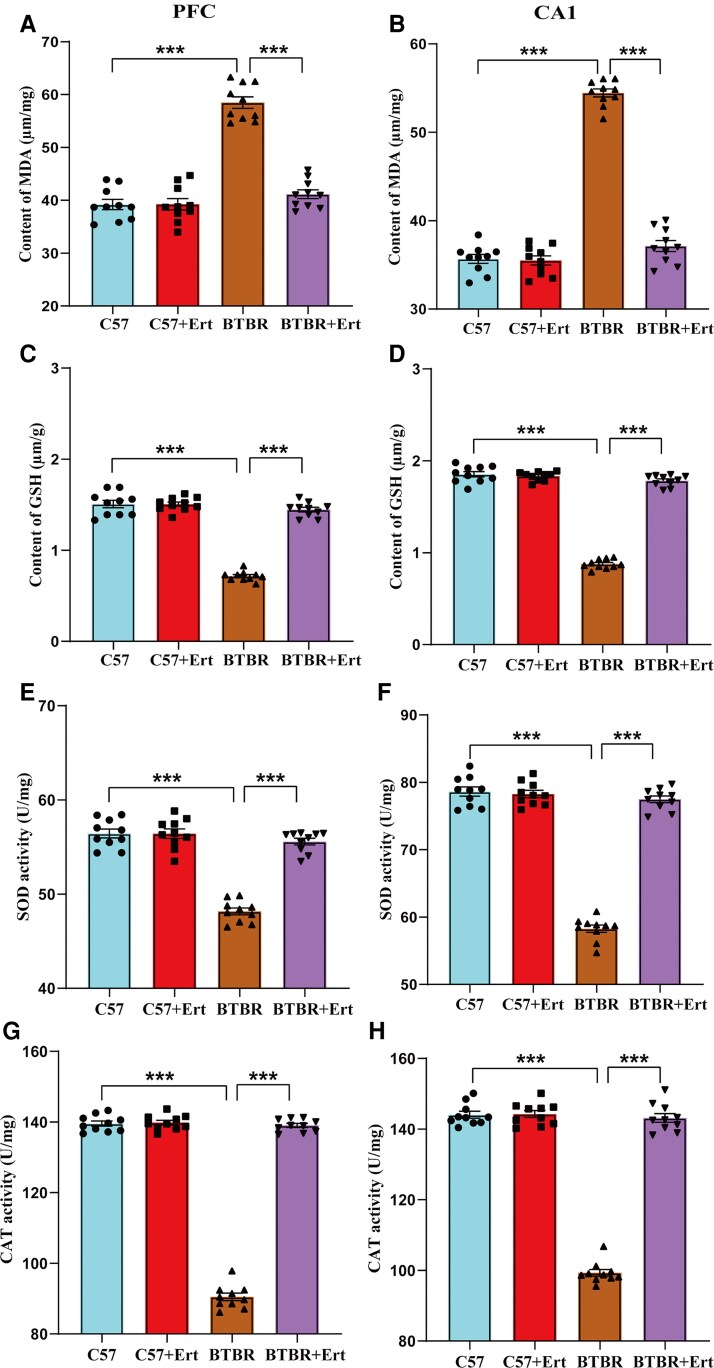
**Ertugliflozin mitigates oxidative stress in the PFC and hippocampus of BTBR mice**. Levels of MDA (**A**), GSH (**C**), SOD (**E**), and CAT (**G**) activity in the PFC. Corresponding measurements of MDA (**B**), GSH (**D**), SOD (**F**), and CAT (**H**) in the hippocampal CA1 region. Data are given as mean ± SEM, with each point indicating an individual mouse (*n* = 10, measured in triplicate). Data were analysed by two-way ANOVA, followed by Tukey's *post hoc* test. ***P  *<* 0.001, BTBR versus C57; ^###^*P*  *<* 0.001, for BTBR + Ert versus BTBR mice.

It is commonly accepted that lipid peroxidation is implicated in neuronal injury in BTBR mice.^34^ BTBR mice were found to have significantly higher MDA levels in the PFC and hippocampus relative to C57 mice (*P* < 0.001), as shown in Fig. 4A-B. However, the administration of ertugliflozin reduced MDA levels relative to the BTBR group (both *P* < 0.001). Furthermore, compared to the C57 group, the BTBR group demonstrated greater oxidative stress, as seen by significantly lower GSH levels and higher SOD and CAT activity in the PFC and hippocampus (all *P* < 0.001; Fig. 4C-H). Ertugliflozin treatment significantly elevated GSH content and the activities of SOD and CAT in the assessed brain regions (all *P* < 0.001; Fig. 4C-H). As a result, these data suggested that prolonged dosing with ertugliflozin substantially reduced oxidative stress in BTBR mouse brains.

### Ertugliflozin suppresses neuroinflammation in the BTBR mouse brain

**Figure 5:**
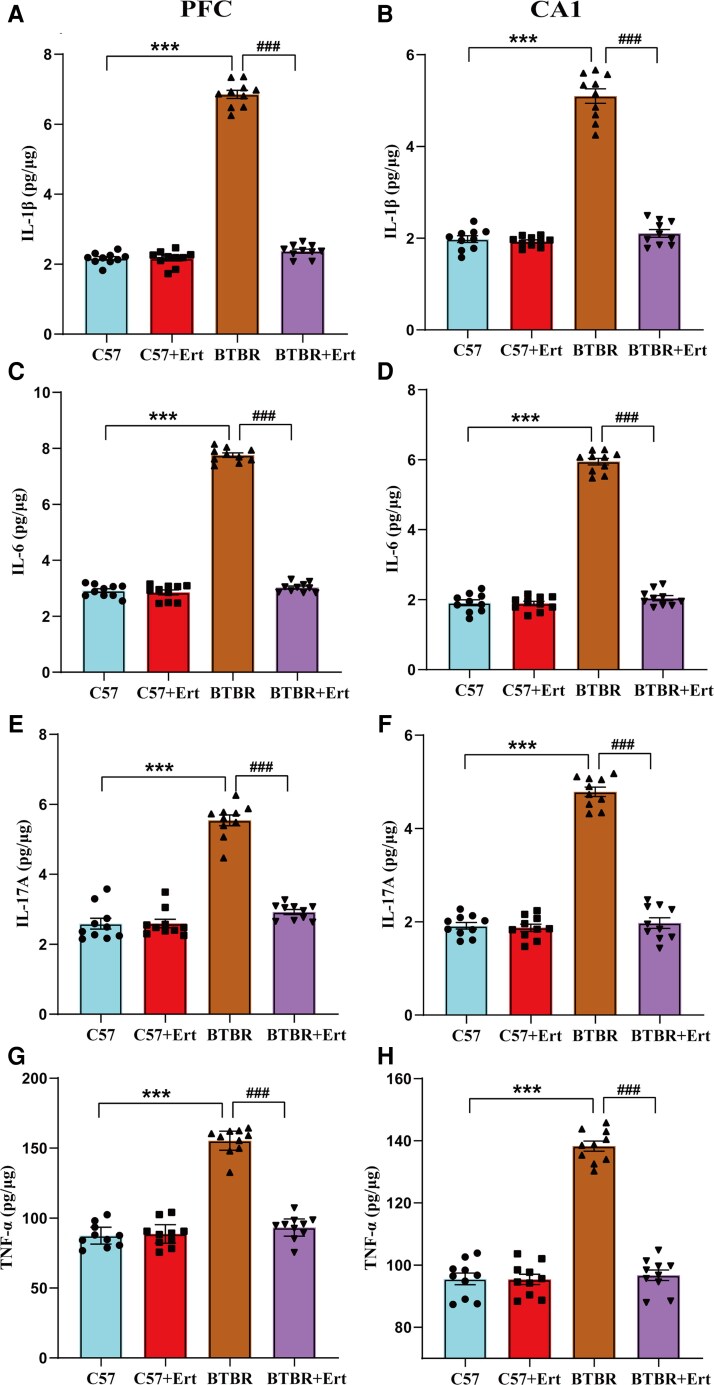
**Effects of ertugliflozin on inflammatory markers in the PFC and hippocampus of BTBR mice.** Levels of IL-1β (**A**), IL-6 (**C**), IL-17A (**E**) and TNF-α (**G**) in the PFC. Corresponding protein levels of IL-1β (**B**), IL-6 (**D**), IL-17A (**F**) and TNF-α (**H**) in the hippocampal CA1 region. Data are given as mean ± SEM, with each point indicating an individual mouse (*n* = 10, measured in triplicate). Data are statistically analysed two-way ANOVA followed by Tukey’s *post hoc* test. ***P  *<* 0.001, BTBR versus C57; ^###^*P*  *<* 0.001, for BTBR + Ert *versus* BTBR group.

Growing evidence supports the role of dysregulated neuroinflammation in autism spectrum disorder.^35-37^ We assessed each mouse’s levels of inflammatory markers to investigate how ertugliflozin therapy affected the pro-inflammatory response in BTBR mice. In the PFC and hippocampus of BTBR mice, levels of inflammatory cytokines IL-1β, IL-6, IL-17A and TNF-α were substantially raised relative to those seen in the controls (Fig. 5A-H; all *P* < 0.001). Treatment with ertugliflozin reduced these levels substantially relative to the BTBR group (all *P* < 0.001). Overall, these findings confirm that ertugliflozin diminishes inflammation in BTBR mice.

### Inhibitory effects of ertugliflozin on Iba1-positive microglia in BTBR mice

**Figure 6:**
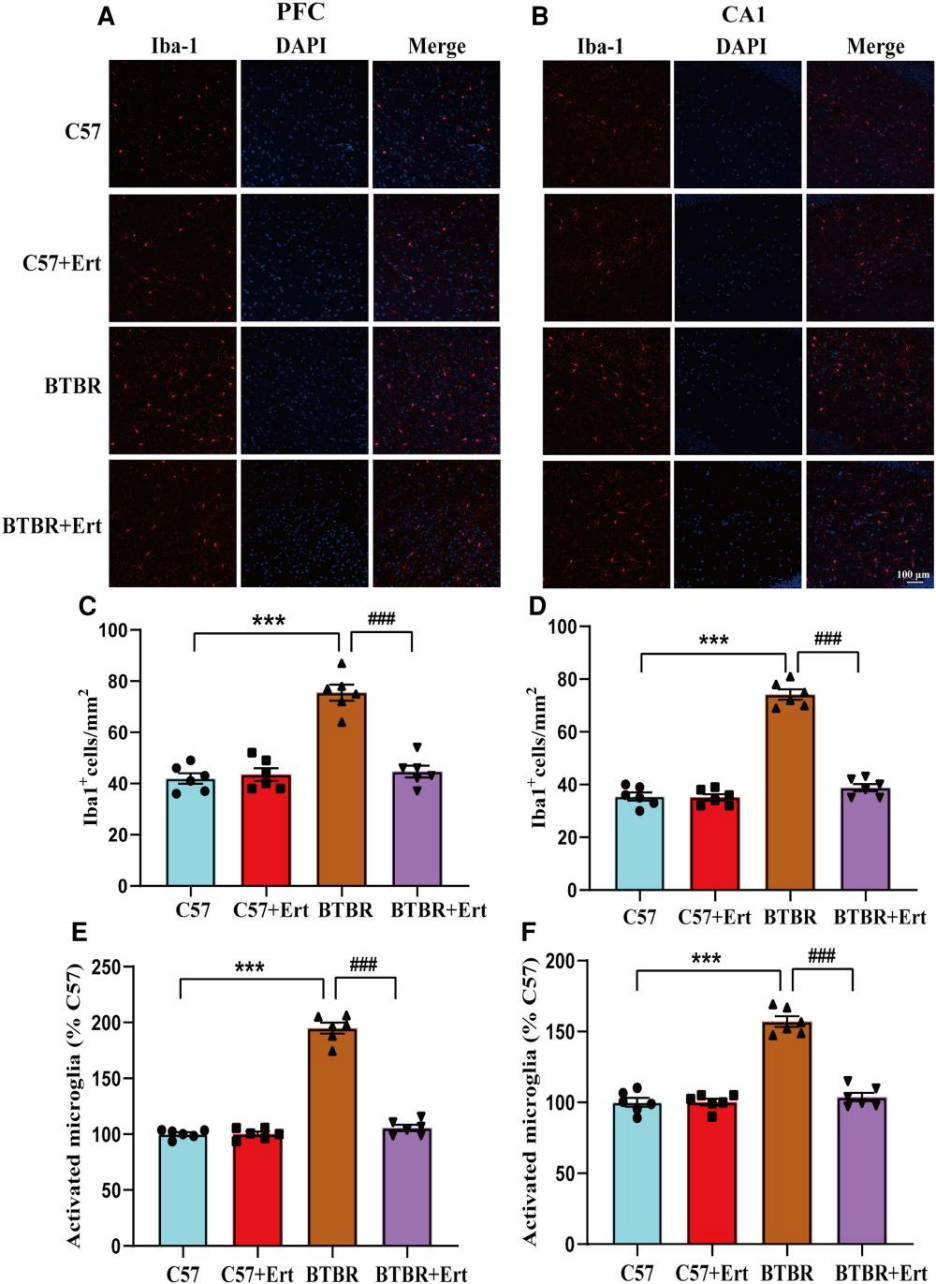
**Ertugliflozin reduces microglial activation in BTBR mice.** Representative immunofluorescence images of microglia in the PFC (**A**) and hippocampal CA1 region (**B**) stained with Iba1 antibody (red), with nuclei counterstained by DAPI (blue). (**C**, **D**) Quantitative analysis of total microglial cell density. (**E**, **F**) The percentage of activated microglia. Results are expressed as mean ± SEM. Each data point represents one mouse (*n* = 6 per group). Data are statistically analysed by two-way ANOVA followed by Tukey’s *post hoc* test for panels. ***P < 0.001 compared with C57 mice; ^###^*P* < 0.001 compared with BTBR mice.

Iba1 is an antigen expressed in microglia.^38^ Microglial cells were identified using Iba1 immunolabelling. As illustrated in Fig. 6A-F, BTBR mice showed a significantly higher density of activated microglia (cells/square millimetre), along with increased activation ratios, in both the PFC and hippocampal CA1 region compared with C57 control mice (all *P* < 0.001). In comparison, ertugliflozin administration significantly reversed these elevations, restoring microglial numbers and activation levels towards those observed in control animals (all *P* < 0.001). Collectively, these findings indicate that ertugliflozin effectively suppresses microglial activation in the hippocampus and PFC of BTBR mice.

## Discussion

Alterations in brain oxidative stress levels and neuroinflammation constitute primary aetiological factors underlying autism spectrum disorder-like symptoms.^39-41^ Converging preclinical evidence indicates that the SGLT2 inhibitor ertugliflozin has promising potential to attenuate these pathological processes.^15,17,42^ This study investigates the therapeutic potential of ertugliflozin to attenuate autism spectrum disorder-like features in BTBR mice while concurrently elucidating its underlying mechanisms of action. Here, we demonstrated that ertugliflozin administration effectively reversed autism spectrum disorder-associated behavioural abnormalities in BTBR mice, including decreased social interactions and elevated repetitive and anxiety-like behaviours. Further analyses indicated that the beneficial effects of ertugliflozin are closely linked to its ability to attenuate oxidative stress and neuroinflammatory responses. All of these findings point to ertugliflozin as a potentially effective treatment for reducing autism spectrum disorder-related symptoms.

Autism spectrum disorder symptoms include difficulties in social behaviours.^43,44^ Numerous lines of evidence show that BTBR mice have communication and social interaction deficiencies that are similar to the main signs of autism spectrum disorder in people.^5,31,45,46^ Previous studies have shown that the SGLT2 inhibitor canagliflozin can improve sociability and social preference induced by sodium valproate (VPA).^47^ Our findings indicated that ertugliflozin therapy improved sociability in BTBR mice, as seen in the marked preference for the unfamiliar animal in the social approach test. Collectively, these findings indicate the presence of anomalies of social interaction that are reversed by ertugliflozin treatment.

It is generally acknowledged that repetitive and stereotyped behaviours are key to autism spectrum disorder diagnostic features.^48^ Numerous studies demonstrate that BTBR mice show greater amounts of marble-burying and self-grooming.^9,12,42,49,50^ It is becoming increasingly evident that canagliflozin enhances stereotypic behaviours in VPA-exposed mice.^47^ Likely, Nakhal *et al*. reported that pre-treatment with canagliflozin reduced shredding in the nestlet-shredding test in VPA-treated rats.^19^ Consistent with these observations, ertugliflozin administration normalized marble-burying behaviour in BTBR mice. Moreover, people with autism spectrum disorder commonly show heightened vulnerability to anxiety-related symptoms.^51^ Pre-treatment with canagliflozin reversed anxiety levels when compared with the VPA mice.^19^ Consistent with earlier reports, our results demonstrate that ertugliflozin effectively alleviates repetitive and stereotyped behaviours as well as anxiety-like features, indicating its ability to restore multiple behavioural phenotypes.

The pathophysiology of autism spectrum disorder is intimately linked to the PFC and hippocampus,^12,52^ and recent research has validated morphological abnormalities in the PFC and hippocampal tissue from BTBR.^53^ In BTBR mice, fewer Nissl bodies and a sparse neuronal distribution were observed in the PFC and hippocampal CA1 area. Also, Nissl staining demonstrated a widespread loss of hippocampus neurons in BTBR animals.^12,49^ Treatment with canagliflozin significantly counteracted histopathological changes in the cerebellum tissues of VPA-treated animals.^47^ In individuals with Type 2 diabetes and Stage 3 chronic kidney disease, ertugliflozin was linked to a decrease in the tubular damage marker KIM-1.^54^ In line with prior results, we found ertugliflozin can prevent neuronal damage in the PFC and hippocampus, as manifested by improvements in neuronal morphology. Therefore, the current study also suggested that treating autism spectrum disorder-like behavioural abnormalities with ertugliflozin could prevent neuronal damage.

Experimental research indicates that oxidative stress plays a crucial role in generating neuronal damage associated with autism spectrum disorder.^41,49,55^ One of the leading causes of behavioural abnormalities in autism spectrum disorder is increased oxidative stress.^49,56^ Using the BTBR mouse model, the current study examined the underlying antioxidant neuroprotective mechanisms of ertugliflozin. An increasing amount of research on post-mortem autism spectrum disorder brains has shown elevated levels of oxidative stress.^15,57,58^ We discovered that ertugliflozin reduces oxidative stress by increasing GSH, SOD and CAT levels in the PFC and hippocampus of BTBR mice. Also, empagliflozin decreased MDA levels in the brain regions of the animal model for autism spectrum disorder. It is well acknowledged that SGLT2 inhibitors may alleviate behavioural and neurobiological abnormalities in autism spectrum disorder patients by reducing brain oxidative stress, which is consistent with our findings.^15^ Specifically, canagliflozin mitigated oxidative stress in a valproic acid-induced autism spectrum disorder rat model as evidenced by decreased MDA levels and restored GSH/CAT activity.^19^ Administration of empagliflozin reduced infarct volume and suppressed cerebral oxidative stress in the rat brain tissue.^18^ Based on this logic, we deduce that ertugliflozin therapy improves antioxidant capacity and lowers oxidative stress in the BTBR mouse brain tissue.

Oxidative stress promotes inflammatory processes and is considered a key factor underlying the behavioural abnormalities observed in autism spectrum disorder.^41,59^ Oxidative stress can drive inflammation in the brain, thereby triggering neuronal damage.^41^ Higher inflammatory activity is frequently found in autistic children.^60,61^ Individuals with autism spectrum disorder have higher levels of IL-6 and IL-1β in their brains, which can lead to altered behavioural patterns.^3,42^ Earlier research found higher levels of TNF-α and IL-17A in BTBR mice’s brain tissue.^39,40,46^ TNF-α regulates gene expression during inflammation. Elevated TNF-α levels cause systemic inflammation.^49^ Furthermore, SGLT2 inhibitors have been demonstrated to attenuate inflammation, including reductions in pro-inflammatory cytokines.^15,17^ Our study found that ertugliflozin reduced overproduction of these factors in the PFC and hippocampus of BTBR mice, suggesting that it may help minimize neuroinflammation in autism spectrum disorder. While empagliflozin has been shown to attenuate inflammation by inhibiting the JAK2/STAT1 pathway in macrophages,^17^ additional studies are needed to determine whether this mechanism explains ertugliflozin’s amelioration of inflammation associated with core autism spectrum disorder-like behavioural characteristics.

Accumulating evidence has confirmed an essential connection among inflammation, immune dysfunction and autism spectrum disorder.^62,63^ In the central nervous system, neuroinflammation involving microglia is recognized.^9^ It was accepted that microglia are the primary neuroimmune cells involved in neuroinflammatory responses in autism spectrum disorder.^22^ Many studies have demonstrated the overactivation of microglia in the post-mortem brains of individuals with autism spectrum disorder,^57,64,65^ suggesting the basic features of neuroinflammation in autism spectrum disorder.^59^ Microglia-driven neuroinflammation associated with autism spectrum disorder-like behavioural abnormalities has been well reported in animal models.^46^ Stephenson *et al*. found that microglia show altered orientation in forebrain areas of BTBR mice.^53^ Bumetanide selectively targeted mPFC microglia and was sufficient to mitigate social novelty impairments in BTBR mice.^66^ Morphological analysis revealed microglial selective activation in the CA3 area of the hippocampus in BTBR mice.^35^ Accordingly, we find that the PFC and CA1 regions of the hippocampus in BTBR mice contain more Iba1-positive cells, indicating activated microglia. Canagliflozin, an SGLT2 inhibitor, protected BV-2 microglia from the inflammatory damage caused by hyperglycaemia.^67^ Overall, our research demonstrated that ertugliflozin therapy deactivated microglia and reduced pro-inflammatory cytokine expression in BTBR animals.

However, the current study has several limitations. First, given the short-term intervention with ertugliflozin, subsequent investigations should assess the persistence of therapeutic effects after discontinuation and the long-term safety profile during sustained administration. Secondly, an imbalance of oxidative stress and inflammatory mediators is associated with autism spectrum disorder-like behaviours. Moreover, oxidative stress activates transcription factors, including PPARγ, Nrf2, HIF-1α and NF-κB, which upregulate pro-inflammatory cytokines.^15^ Whether these transcription factors mediate ertugliflozin’s alleviation of oxidative stress- and inflammation-associated autism spectrum disorder-like phenotypes remains to be elucidated. Thirdly, male animals were solely included in this investigation. Divergence between male and female parameters is not addressed. Future research should examine the impacts on both sexes because oxidative stress and inflammation may have different effects on males and females with autism spectrum disorder.

In conclusion, our findings show that in the BTBR mouse model of autism spectrum disorder, ertugliflozin treatment significantly reduces anxiety, limited and repetitive behaviours and social interaction deficits. The results obtained further suggest that ertugliflozin may suppress oxidative stress, inflammation and microglial overactivation as part of its mechanism of action. The findings of this investigation broaden our knowledge of ertugliflozin’s enormous therapeutic potential for autism spectrum disorder and may provide fresh perspectives on the treatment of neuropsychiatric disorders. To confirm its effectiveness and safety, numerous preclinical studies and comprehensive clinical trials are essential.

## Data Availability

The data that support the findings of this study are available from the corresponding author upon reasonable request.
