# Correction to: Ertugliflozin improves animal behaviours associated with oxidative stress and inflammation in a BTBR *T* + *Itpr3tf/J* mouse model of autism

**DOI:** 10.1093/braincomms/fcag213

**Published:** 2026-06-05

This is a correction to: Xiaona Wang, Zhengqin Zhao, Limin Sun, Chao Gao, Li Wang, Daoqi Mei, Chanjuan Hao, Shuai Zhao, Xingxue Yan, Jing Liu, Lei Liu, Bin Guo, Yaodong Zhang, Ertugliflozin improves animal behaviours associated with oxidative stress and inflammation in a BTBR *T* + *Itpr3tf/J* mouse model of autism, *Brain Communications*, Volume 8, Issue 2, 2026, fcag083, https://doi.org/10.1093/braincomms/fcag083

In the originally published version of this manuscript, an error was present in affiliation 1. The affiliation was originally listed as follows:

“Henan Key Laboratory of Genetic and Developmental Disorders, Henan Children's Neurodevelopment Engineering Research Center, Henan Academy of Medical Sciences, Children's Hospital Affiliated to Zhengzhou University, Henan Children's Hospital, Institute of Children's Health, Zhengzhou 450018, China”.

The affiliation has now been corrected in the published article to read:

“Henan Key Laboratory of Genetic and Developmental Disorders, Henan Children's Neurodevelopment Engineering Research Center, Children's Hospital Affiliated to Zhengzhou University, Henan Children's Hospital, Institute of Children's Health, Henan Academy of Medical Sciences, Zhengzhou, 450018, China”.

